# The effects of second-hand smoke on biological processes important in atherogenesis

**DOI:** 10.1186/1471-2261-7-1

**Published:** 2007-01-08

**Authors:** Hongwei Yuan, Lina S Wong, Monideepa Bhattacharya, Chongze Ma, Mohammed Zafarani, Min Yao, Matthias Schneider, Robert E Pitas, Manuela Martins-Green

**Affiliations:** 1 Graduate Program in Cell, Molecular and Developmental Biology, University of California, Riverside, Riverside, California, USA; 2 Department of Cell Biology and Neuroscience, University of California, Riverside, Riverside, California, USA; 3 Gladstone Institute of Cardiovascular Disease, University of California, San Francisco, San Francisco, California, USA

## Abstract

**Background:**

Atherosclerosis is the leading cause of death in western societies and cigarette smoke is among the factors that strongly contribute to the development of this disease. The early events in atherogenesis are stimulated on the one hand by cytokines that chemoattract leukocytes and on the other hand by decrease in circulating molecules that protect endothelial cells (ECs) from injury. Here we focus our studies on the effects of "second-hand" smoke on atherogenesis.

**Methods:**

To perform these studies, a smoking system that closely simulates exposure of humans to second-hand smoke was developed and a mouse model system transgenic for human apoB^100 ^was used. These mice have moderate lipid levels that closely mimic human conditions that lead to atherosclerotic plaque formation.

**Results:**

"Second-hand" cigarette smoke decreases plasma high density lipoprotein levels in the blood and also decreases the ratios between high density lipoprotein and low density lipoprotein, high density lipoprotein and triglyceride, and high density lipoprotein and total cholesterol. This change in lipid profiles causes not only more lipid accumulation in the aorta but also lipid deposition in many of the smaller vessels of the heart and in hepatocytes. In addition, mice exposed to smoke have increased levels of Monocyte Chemoattractant Protein–1 in circulation and in the heart/aorta tissue, have increased macrophages in the arterial walls, and have decreased levels of adiponectin, an EC-protective protein. Also, cytokine arrays revealed that mice exposed to smoke do not undergo the switch from the pro-inflammatory cytokine profile (that develops when the mice are initially exposed to second-hand smoke) to the adaptive response. Furthermore, triglyceride levels increase significantly in the liver of smoke-exposed mice.

**Conclusion:**

Long-term exposure to "second-hand" smoke creates a state of permanent inflammation and an imbalance in the lipid profile that leads to lipid accumulation in the liver and in the blood vessels of the heart and aorta. The former potentially can lead to non-alcoholic fatty liver disease and the latter to heart attacks.

## Background

Atherosclerosis is an inflammatory disease that accounts for nearly 50% of deaths in western societies [1]. Initiation of atherosclerotic plaque formation is a complex process. It involves secretion of chemokines such as the Monocyte Chemoattractant Protein–1 (MCP-1) [1-4] and expression of adhesion molecules on the surface of monocytes and endothelial cells [5-7]. Circulating monocytes are recruited to sites of injured endothelial cells, adhere to them, and migrate into the subendothelial space. Monocytes in the arterial wall differentiate into activated macrophages that are efficient scavengers of oxidized low density lipoprotein (LDL). When exposed to large amounts of oxidized LDL, these macrophages accumulate large amounts of cholesteryl esters in lipid droplets and become "foam cells" that form "fatty streaks", the precursors of more complicated atherosclerotic plaques [8,9].

Many factors can lead to initiation of atherosclerosis. In the Framingham heart study [10-12], the best-known prospective investigation that established the risk factors for coronary heart disease and peripheral vascular disease, smoking was identified as one of the major risk factors for the development of atherosclerosis. Cigarette smoke is a complex mixture of more than 4,700 chemical constituents distributed in particulate and gaseous phases, including nicotine, aromatic hydrocarbons, sterols and oxygenated isoprenoid compounds, aldehydes, nitriles, cyclic ethers, and sulfur compounds [13]. Cigarette smoking accelerates atherosclerosis in the coronary arteries, the aorta, the carotid and cerebral arteries, and the large arteries in the peripheral circulation. Toxic substances present in cigarette smoke build up in the areas of curvature and branching of arteries, injuring the endothelium [2,14]. Furthermore, epidemiological studies have shown that both active and passive cigarette smoking increase the risk of atherogenesis [9]. In spite of all the evidence that cigarette smoke stimulates atherogenesis and lipoprotein oxidation may play an important role [9], very little is known about the biological processes induced by smoke that contribute to increased cardiovascular disease.

Chemokines are small (8–10 kDa) stress-response proteins expressed when organisms or cells are exposed to an insult. The effects of cigarette smoke on atherosclerosis are no exception. MSW (mainstream whole, "first-hand") smoke and SSW (sidestream whole, major component of "second-hand" smoke) smoke recently have been shown to stimulate human fibroblasts to express several chemokines, including MCP-1 [15-17]. This chemokine plays a key role in atherosclerotic lesion formation [18-23]. However, studies to determine the effects of cigarette smoke on atherosclerosis have been hampered because of the lack of a stress-free system to expose the mice to smoke. In most of the published studies, when the animals were exposed to smoke, they were confined to a small device without free access to food and water. This invariably put the animals under added stress in addition to that caused by the cigarette smoke [24]. Therefore, for these studies we developed a smoking system that allows the animals being exposed to cigarette smoke to move freely and consume water and food at will. Also important, with this system we can separate and control SSW and MSW exposure. In addition, this system allows for control of smoke dosage.

The most commonly used mouse model systems for studies of atherogenesis are mice deficient in either apolipoprotein E (ApoE -/-) or the low density lipoprotein receptor (LDLR-/-). Whereas these models have the advantage of developing lesions in a short time and are easy to manage, lesion formation occurs under altered physiological conditions, such as hyperlipidemia. The LDLR-/- mice represent a genetic defect that leads to hypercholesterolemia. These mice are excellent to model disease conditions but do not represent well the most common situation in humans, i.e. formation of atherosclerotic lesions under moderate lipidemia. Therefore, to study the effects of SSW on biological processes involved in initiation of plaque formation, human apoB100 transgenic mice [25] fed a high fat diet were used. These animals have moderate hypercholesterolemia, much like most humans that develop atherosclerosis. Our results show that many of the early indicators of plaque initiation are stimulated by SSW in these mice and suggest a mechanism by which SSW accelerates atherogenesis.

## Methods

### Key reagents

All common chemicals were purchased from Sigma Biochemicals and Fisher Scientific; LDL L-Type test, L-Type HDL-C test, Colesterol C II test, and L-Type TG H test (Waco Diagnostics, Richmond, VA); Tissue-Tek Optimal cutting Temperature (OCT)(Sakura Finetek USA, Torrance, CA); Oil-Red-O (Fisher Scientific). Primary antibodies: Rabbit anti-mouse-MCP-1 (Cell Sciences, Canton, MA); rat anti-mouse F4/80 (Serotec, Oxford, UK); rabbit anti-human myeloperoxidase which cross-reacts with mouse) (Biomeda, Foster City, CA); goat anti-mouse adiponectin (R&D Systems Minneapolis, MN). Secondary antibodies: chicken anti-rat-Alexa488 (Gift from Dr. Christian Lytle); goat anti-rabbit-FITC (Zymed, South San Francisco, CA); rabbit anti-goat conjugated to HRP (Rockland immunochemicals, Inc., Gilbertsville, PA); goat anti-rabbit conjugated to HRP for subsequent use with the Super signal West Dura extended duration substrate (Pierce Chemical, Rockford, IL); ECL detection system (Amersham Piscataway, NJ); Vectashield mounting medium (Vector Laboratories, Burlingame, CA); DC protein assay kit (Bio-Rad, Hercules, CA).

### Animal system and diet

6–8 week old male mice (57BL/6Ntac-TgN(APOB100)) were purchased from Taconic Farms, Inc. [25]. 6–8 week old male apoB100 transgenic mice (apoB100 Leu-Leu) on 57BL/6SJL background were also used. The latter mice differ from the former in that they over-express human apoB100 without expression of apoB48, due to a mutation in codon 2163 that converted CAA the codon for Gln to CTA the codon for Leu effectively precluding the expression of apoB48 [26]. The mice were maintained in a pathogen-free facility with a 12-hrs light/12-hrs dark cycle and had free access to water and food. The mice were kept on regular diet to adapt to the environment for one week, and then changed to a high fat diet. This diet contains 1.25% Cholesterol, 15% cocoa butter, and 0.5% sodium cholate [27,28] and was purchased from MP Biomedicals, Inc. The mice were put into the smoking experiments a week after switching them to the high fat diet. All animal experiments were conducted in accordance with U.S. Public Health Service/U.S. Department of Agriculture guidelines. Experimental protocols were approved by the University of California Riverside Institutional Animal Care and Use Committee.

### Exposure of the animals to smoke

The cigarettes used in this study (1R3F research grade) were purchased from Kentucky Tobacco Research & Development Center. A puffer box was used to generate smoke according to FTC guidelines: 1 puff/minute, 3 seconds/puff. Each smoke type was mixed with fresh air before it was puffed through a tube into the small mouse exposure chambers (Teague Enterprise). The air in the chambers was exhausted using a fan (vortex blower). The mice in the smoking groups were exposed to the smoke for 6 hrs/day (10 min smoking with 5 min break), 5 days/week. Smoke particle concentration in the chamber was maintained at 25 ± 2 mg/m^3 ^by adjusting the fresh air entering and exhausting from the chamber. Smoke samples taken from each chamber were passed through a filter; the particulate material deposited on the filter was measured by weighing the filter before and after smoke filtration. The vacuum pump gas meter recorded the smoke sample volume passed through the filter. Total particulate matter (TPM) was calculated by dividing the total particle weight trapped on the filter by the volume passed through the filter.

### Smoke exposure analysis

Inhalation of smoke by the mice was monitored by measuring the blood carboxyhemoglobin level according to Griffith et al. [24]. Briefly, 20 μl of blood from the mouse was diluted to 20 ml with 0.04% ammonium hydroxide. The absorbance values at 413 nm and 420 nm were determined and the 420/413 ratio was calculated. An increase in this ratio reflects smoke exposure. We also measured and calculated the blood carboxyhemoglobin level. Briefly, 10 μl of blood were mixed with freshly prepared sodium dithionite (2 mg/ml) in 0.01 mol/l TRIS (hydroxymethyl) aminomethane solution, and absorption spectra at 420 and 432 nm were recorded within 2–3 minutes. The concentration of COHb was calculated using the following equation: Percent COHb = 100 × [1 - (Ar × F1)]/[Ar × (F2 - F1) - F3 + 1], where Ar = Abs420 nm/Abs432 nm, F1 = 1.3007, F2 = 0.4648, F3 = 2.0526 according to previously published work [29,30].

### Plasma lipid analysis

Levels of total cholesterol (TC), triglycerides (TG), low density lipoproteins (LDL), and high density lipoproteins (HDL) in plasma were determined by enzymatic assay using commercially available kits (Wako Chemical Co.) and following the instructions given by the company.

### Tissue lipid analysis

After the mouse was sacrificed, its liver was harvested and frozen immediately on dry ice and kept at -72°C. Lipids were extracted from about 300 mg liver tissue (wet weight) into 30 ml chloroform: methanol (2:1) according to the method of Folch *et al*. [31]. Phases were separated by addition of 6 ml 0.05% H_2_SO_4 _and aliquots of extracted lipids were mixed with 1% Triton X-100, dried under a stream of N_2_, and re-dissolved in water [32]. Commercial kits were used for the specific determination of total cholesterol and triglycerides (WAKO Chemicals USA, Inc., Richmond, VA). The hepatic lipid concentrations were expressed as milligrams of lipids per gram of wet weight.

### Lesion analysis

Mice were fasted for 4 hrs, anesthetized with ketamine and xylazine and were perfused with 10–15 mL of PBS, pH 7.4 (80 mmHg), followed by 4% paraformaldehyde for 3–5 minutes [21]. The heart and aorta were removed and fixed for an additional 1–2 hrs in 4% paraformaldehyde followed by infiltration with 30% gum sucrose (1% gum Arabic, 30% sucrose in PBS) for 24 hrs at 4°C and embedded in OCT for sectioning [33]. Briefly, the OCT-embedded, frozen aortas were cross-sectioned serially at 10-μm thicknesses for a total of 300 μm beginning at the base of the aortic valve, where all three leaflets are first visible. Every fourth section for a total of five sections from each animal was stained with Oil-Red O (Fisher scientific company) and counterstained with hematoxylin (Sigma) to identify the lipid-rich lesions. Average lesion sizes of these 5 cross sections were used for morphometric evaluations. Images were captured using a Nikon microscope with camera and scanned into a computer. Quantitation of atherosclerotic lesions was performed using NIH Image software. Results are expressed as percent of the total cross-sectional vessel wall area [21].

### Immunolabeling

Sections of the heart and aorta were used for immunostaining for MCP-1 with a primary rabbit anti-mouse polyclonal antibody (Cell Sciences). As secondary antibody, a FITC-labeled goat anti-rabbit IgG was used. For macrophage staining, rat anti-mouse F4/80 antibody (Serotec, undiluted) was used as primary antibody and Alexa Fluor 488-labeled chicken anti-rat antibody was used as secondary antibody. For neutrophil staining, rabbit anti-MPO antibody was used as primary antibody and FITC-labeled goat anti-rabbit IgG was used as secondary antibody. For splenic cell staining, rat anti mouse CD4+, IL-4, or IFNγ antibodies (BioLegend) were used as primary antibodies and FITC-labeled goat anti-rat IgG was used as secondary antibody. Quantitation was performed by counting the labeled cells in 3 high-power fields/section of spleen, taking the average and then calculating the ratio. In cell clusters, the number of stained cells was estimated by the size of the cells.

### Cytokine antibody array

Cytokine levels in the plasma were measured using Q-Plex™ Mouse Cytokine array (Quansys Biosciences, Utah, USA), which is a fully quantitative ELISA-based test in which each well of a 96 well plate was coated with 16 distinct capture antibodies in a defined array. 30 μl of cytokine standard or plasma sample were added into the appropriate wells. After 1 hr incubation, the wells were washed 3 times and probed with streptavidin-HRP for 15 minutes at room temperature. After washing 3 times and adding the substrate, the images were captured by a cooled CCD camera (MicroMAX-1300B, Roper Scientific) and analyzed using the Quansys image analysis software. The concentrations of cytokine were calculated and plotted using the standard curve.

## Results

### Cigarette smoking system and calibration

**Figure 1:**
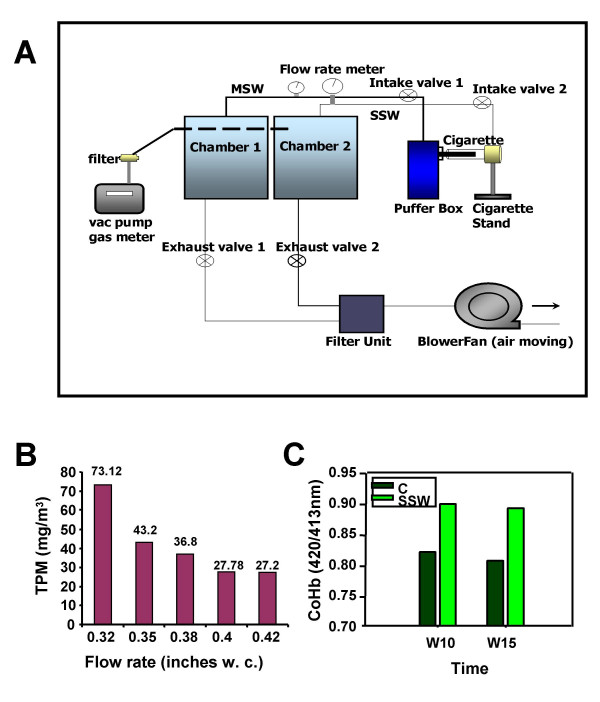
**Schematic representation of the Smoking System, smoking dose calibration, and effects of cigarette smoke on blood carboxyhemoglobin (CoHb) levels**. (**A**) Schematic representation of the smoking system. Smoke was generated by using a puffer box built by the University of Kentucky [33] and chambers built by Teague Enterprises. Each smoke type can be mixed with fresh air before it was puffed through a tube into the exposure chambers. In this study only the SSW chamber was used. The air and smoke in the chamber were exhausted using a blower fan (air moving fan). A vacuum pump/gas meter was used for dose calibration only. (**B**) The smoking dose was calibrated to 25 mg TPM/m^3 ^by adjusting the intake and exhaust valves. Smoke samples taken from the SSW chamber were passed through a filter unit; the particulate material deposited onto the filter was measured by weighing the filter before and after smoke filtration. The vacuum pump gas meter recorded the smoke sample volume passed through the filter. Total particulate matter (TPM) was calculated by dividing the total particle weight trapped on the filter by the volume passed through the filter. w.c. = water column. (**C**) Mice were exposed to SSW smoke for 6 hrs intermittently and blood was drawn to test for carboxyhemoglobin. Blood was treated with ammonium hydroxide, absorbance measured at 420 nm and 413 nm and the ratio plotted. C = control.

A system that can be used to administer cigarette smoke to small animals under conditions that minimize stress was developed (Figure 1A). In this system, the MSW (Mainstream whole, "first-hand") and the SSW (Sidestream whole, a major component of "second-hand") smoke generated from a puffer box are distributed to separate smoking chambers. MSW smoke is defined as the smoke inhaled by the smoker, whereas SSW smoke is defined as the smoke emanating from the burning end of the cigarette. For this study, the mouse cages with food and water are introduced into the chamber that receives only SSW; the mice are free to move around and eat and drink at will. Therefore, the mice are exposed only to SSW, the major component of second hand smoke. The dose of smoke in each chamber is controlled by adjusting the intake and exhaust valves (see Materials and Methods). When making the measurements, the filter unit connected to the vacuum pump gas meter is connected to the smoking chamber, and the smoke sample from the chamber passes through the filter unit. The filter unit holds a special filter paper that can trap particles in the smoke sample. The vacuum pump meter was set to collect sample for 10 minutes, and the volume of the smoke passed through the filter unit can be calculated from the meter readings of the start and end points. The weight of the filter was determined both before and after each sampling; the weight difference of the filter paper is the weight of particulate matter trapped by the filter. The smoking dose was obtained by calculating the weight of particulate matter per cubic meter of sample in the chamber.

Because second-hand smoke exposure occurs in environments that are usually out of control of the person(s) being exposed, e.g. children and teens of households in which the parents smoke, we decided to focus our studies on the effects of SSW on processes that relate to atherogenesis. As in previous studies [34,35], a smoking dose of 25 ± 2 mg/m^3 ^total particulate matter (TPM) was used. The mice were exposed to SSW for 6 hours, with 5 minutes break every 10 minutes, the time taken to smoke 1 cigarette. The total exposure thus is 24 cigarettes/day, which is similar to the average number of cigarettes smoked indoors in a typical smoker's home (an average smoker smokes about 1.4 packs or 28 cigarettes/day [36]). To arrive at the dose of 25 ± 2 mg/m^3^, the chambers were set at a pressure of 0.42 inches w.c. (water column), which equals 0.104 kPa. For the smoking cage used (19 in × 19 in × 16 in), the flow rate at this pressure was 28.1 liter/min. Extra smoke was filtered to reduce air pollution and expelled from the chamber into the fume hood by the blower fan (Figure 1B). To verify that the mice were exposed to cigarette smoke, the blood carboxyhemoglobin (CoHb) level was measured. SSW-exposed animals showed an increased level of blood carboxyhemoglobin, as expected (Figure 1C). COHb levels were significantly higher in mice exposed to CS compared to mice exposed to air (8.11 ± 1.08%, n = 4 versus 2.38 ± 0.16%, n = 6, respectively; *P *< 0.01). The mean COHb concentrations for nonsmokers were reported to be about 1–2% and those for smokers about 4–7% [37,38]. In heavy smokers, the COHb concentration was 12%. In the mice model, the value may be slightly higher, 1.2 ± 0.8% versus 12.2 ± 4.9%, respectively [37]. Therefore, the amount of pulmonary stress to the animals was similar to reported studies.

### SSW causes changes in lipid physiology

**Figure 2:**
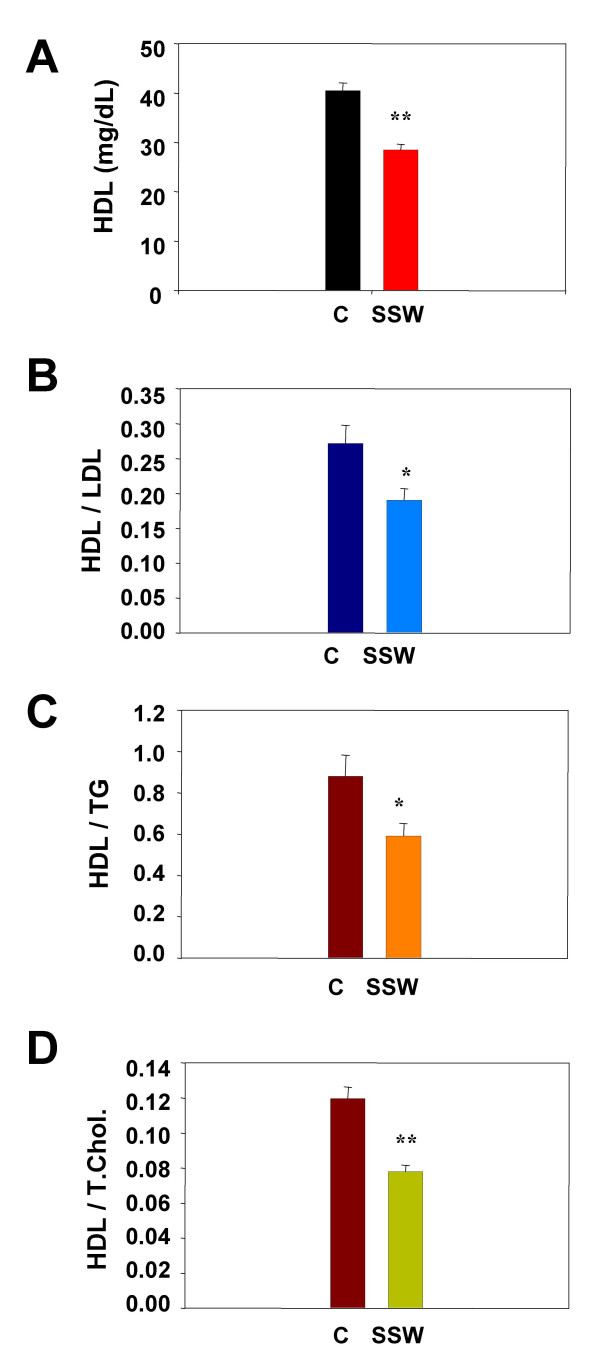
**Effect of cigarette smoke on the HDL and its ratio to other plasma lipids**. Mice were exposed to SSW 6 hrs/day intermittently, 5 days a week. Blood samples were collected from the tail vein and, after centrifugation, the plasma was used to analyze for levels of HDL and other lipids by enzymatic reaction using colorimetric methods with commercially available kits (Wako Chemicals). After incubating with coloring reagent that only reacts with a specific cholesterol or type of lipid, the absorbance was measured and the concentration of each was calculated by plotting the measured values against a standard curve. (**A**) The levels of HDL in the blood of mice exposed to SSW are significantly lower than in the non-exposed mice. The ratios of HDL to LDL (**B**), HDL to TG (**C**), and HDL to TC (**D**) are all significantly lower in the SSW exposed mice.

In patients with cardiovascular disease, the HDL/LDL ratio decreases significantly. A low HDL/LDL cholesterol ratio is an important risk factor for atherosclerosis. HDL participates in reverse cholesterol transport, i.e., it carries cholesterol away from the arteries and other peripheral tissues and back to the liver for excretion from the body. This removes excess cholesterol from plaques, thus minimizing the buildup of cholesterol in the artery walls. SSW smoke decreases the HDL level in the plasma (Figure 2A) and decreases the ratio between HDL and LDL, HDL and triglyceride (TG), and HDL and total cholesterol (TC) (Figure 2B–D). The total plasma cholesterol level is 342.35 ± 29.59 mg/dl for control mice, and 334.72 ± 22.37 mg/dl for SSW-exposed mice, respectively.

**Figure 3:**
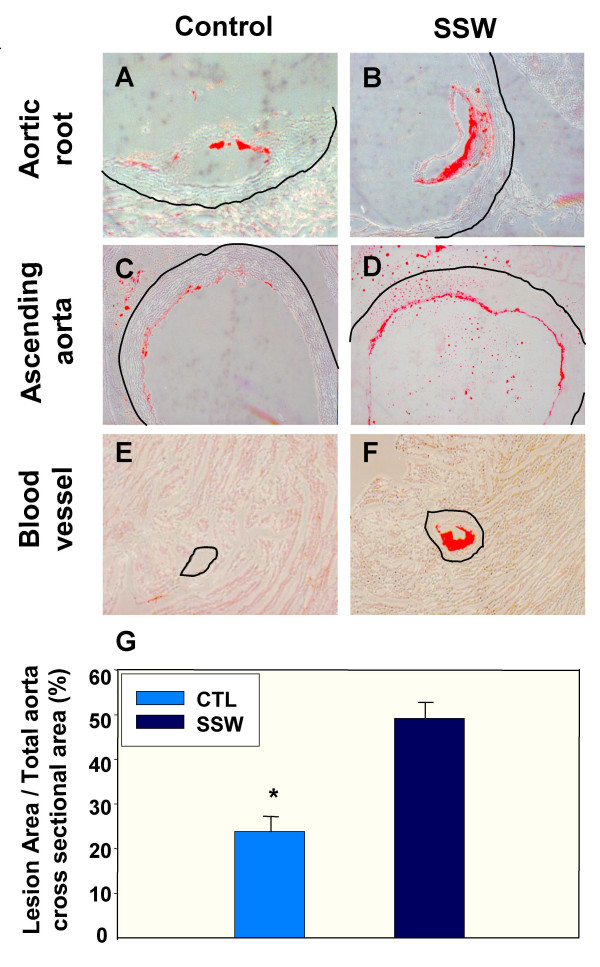
**Lipid accumulation in arterial blood vessels**. Oil red O was used to detect the presence of lipids in tissue sections of the heart and aorta. **(A) & (B) **Presence of higher lipid deposition in the root of the aorta in the smoke-exposed mice. **(C) & (D) **Similar to A&B but in the proximal part of ascending aorta. **(E) & (F) **Small blood vessels in the heart muscle of SSW- exposed mice show much more lipid accumulation than in control mice. Thin black lines outline the outside of the vessel walls. **(G) **The lesion area in both control and SSW-exposed mice was measured as described in the Materials and Methods. The results are expressed as percentage of total area of aorta wall in cross section. Data are mean ± SEM for each group. Statistical analyses were done according to unpaired t test. *P < 0.05 vs. control. The mice were exposed to SSW for 1 year. The images are representative of 2 mice per group.

To determine if the changes in the lipid levels induced by SSW lead to an adverse effect in the hApoB100 transgenic mice (57BL/6Ntac-tgN) [25], Oil-Red-O staining of cross sections of the heart/aorta tissues were performed. SSW-exposed animals did not only accumulate more lipids in the aortic walls (Figure 3A–D), but also had many of the smaller vessels in the heart tissue itself that were almost filled with lipid (Figure 3E, F). The lesion areas measured in the cross sections of the aorta account for 23.83 ± 3.41% and 49.05 ± 3.67% of the total aorta wall areas for control mice and SSW exposed mice, respectively (Figure 3G). To confirm these results, transgenic mice expressing only human apoB100 were used. These latter mice more closely mimic human conditions because they produce human apoB100 that is a constituent of the atherogenic LDL, but not apoB48, a major cholesterol carrier in mice that is normally not considered to associate with atherogenic lipoproteins[26]. The same effects were found: more lipids accumulated in the aortic wall and in the small vessels of the heart after cigarette smoke exposure (data not shown).

**Figure 4:**
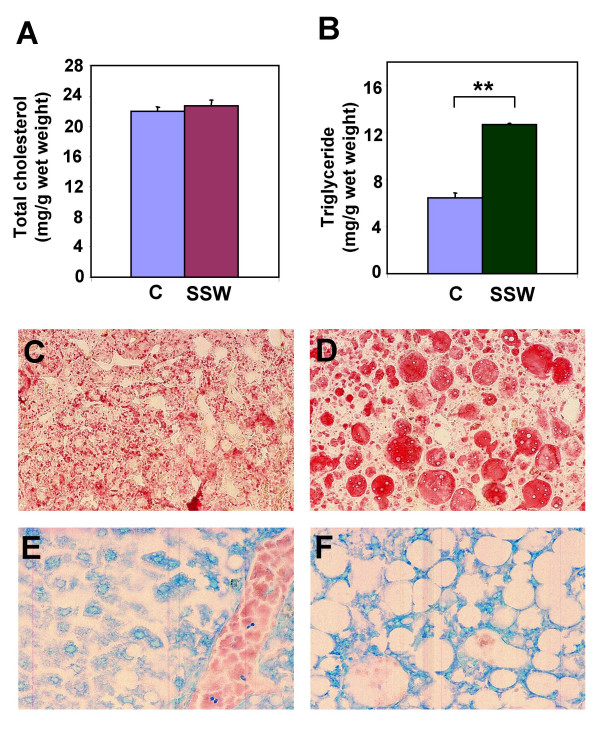
**Lipid accumulation in liver tissue**. About equal amounts of snap-frozen liver from the sacrificed mice of both control and SSW mice were used for lipid extraction with chloroform: methanol (2:1). The extracted lipids were dried and redissolved in 1% Triton X-100. Commercial kits (WAKO Chemicals USA, Inc., Richmond, VA) were used for determination of **(A) **total cholesterol and **(B) **triglycerides. **(C-F) **Oil Red O staining of frozen liver sections reveals the lipid contents in the liver of control mice **(C) **and SSW mice **(D)**. The liver of control mice **(E) **and SSW mice **(F) **were also stained with Diff-Quick to show their histological structure. The mice were exposed to SSW for 1 year. Representative images of one mouse out of three.

The amount of total cholesterol and triglycerides in liver tissue were measured because synthesis and metabolism of lipids occur in the liver. Although there was no significant change in the total cholesterol level (Figure 4A), the triglyceride levels increased significantly after the mice were exposed to SSW (Figure 4B). Oil-Red-O staining of liver sections showed that there was much more lipid accumulation in the liver of mice exposed to SSW (Figure 4D) than in the liver of control mice (Figure 4C). Diff-Quick staining shows that the lipid droplets are much bigger in the SSW smoking mice than in the control mice (Figure 4E &4F).

### Mice exposed to SSW have increased levels of MCP-1 in both plasma and heart/aortic tissues

**Figure 5:**
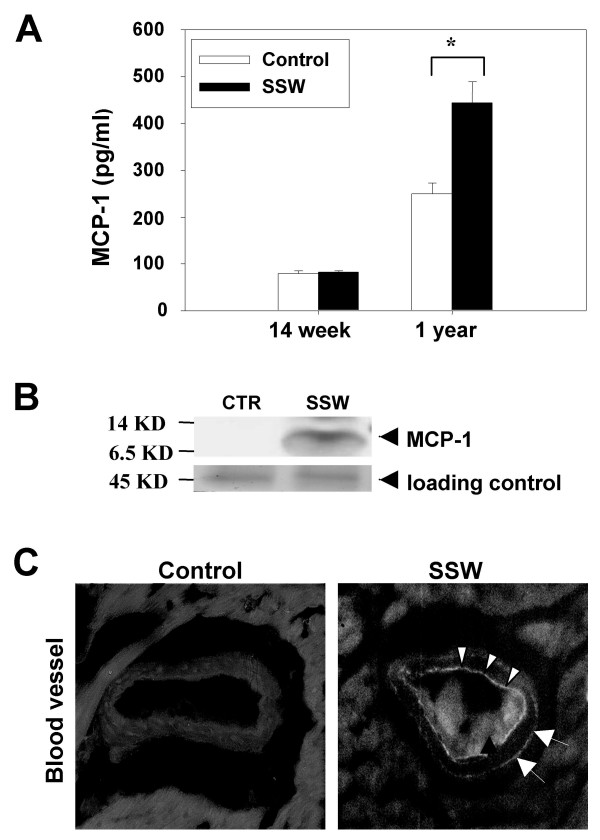
**MCP-1 in the plasma and heart/aortic tissues in human apoB100 transgenic mice on high fat diet and exposed to SSW: **(**A**) Levels of MCP-1 in the plasma of smoking mice at two different time points during smoking. 30 μl of cytokine standard or plasma sample were added into the appropriate wells of a Mouse Cytokine array (Quansys Biosciences, Utah, USA) containing an antibody to this chemokine. The images were captured by a CCD camera and analyzed using the Quansys image analysis software. The concentration of MCP-1 was calculated and plotted using a standard curve. (**B**) Immunoblot analysis for MCP-1 in the heart/aortic tissue (the upper half of the heart together with ascending aorta). The protein extractions were quantified using a DC protein assay kit (Bio-Rad, Hercules, CA) and equal amounts of protein were loaded in each lane of SDS-Polyacrylamide Gel. A 45 KD protein was used to evaluate equal loadings because this protein is always constant, independent of the treatment when the gels are stained with Commasie blue. (**C**): Immunolabeling for MCP-1 in an arterial vessel of the heart tissue shows that this chemokine is produced by the endothelial cells (arrowheads) and by some of the cells in the adventitia (arrow). The mice were exposed to SSW periodically for 1 year. Representative images of one mouse out of three.

MCP-1 has been shown to be a critical participant in atherogenesis because it is a potent chemoattactant for monocytes that infiltrate the intimal layer of arterial walls following injury [1,2,4]. Thus, the levels of MCP-1 in the plasma of SSW-exposed mice were examined and were found that early in the process of smoking the mice have normal MCP-1 levels but by the end of one year of smoking there is a significant increase in the levels of circulating MCP-1 (Figure 5A). The heart/aorta tissues were also examined for the level of MCP-1 protein by immunoblot analysis. These tissues were found to contain higher levels of the chemokine (Figure 5B); immunolabeling showed MCP-1 is primarily localized in the endothelial cells of large vessels (Figure 5C; arrowheads). Some staining was also present in cells of the adventitia (Figure 5C; arrows). These results in apoB100 mice were independent of apoB48 expression.

### Presence of macrophages in the arterial walls of SSW-exposed mice

**Figure 6:**
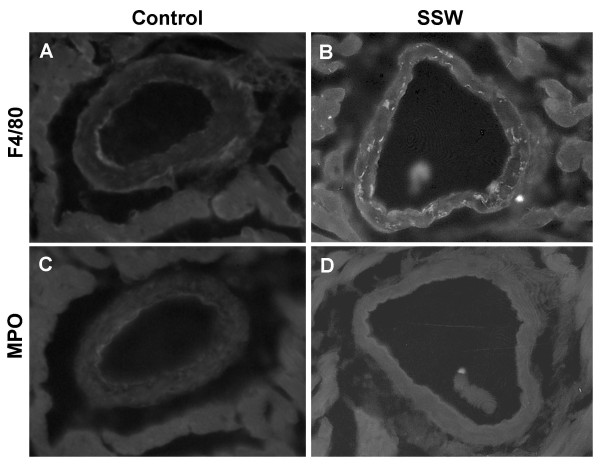
**Macrophages and neutrophils in apoB100 transgenic mice on high fat diet: (A) & (B) **Tissue sections of arterial blood vessels in the heart were immunolabeled with the antibody to F4/80 which is specific to macrophages, and (**C**) & (**D**) with an antibody to myeloperoxidase to detect neutrophils. Macrophages were observed in the walls of arterial vessels in mice exposed to SSW smoke but not in the control mice. However, neutrophils were not found in the arterial vessel walls of either control mice or mice exposed to SSW. The mice were exposed to SSW for 1 year as described in the Materials and Methods. Representative images of one mouse out of three.

The process of atherogenesis is characterized by monocyte migration from the lumen to the intimal (subendothelial) layer of the blood vessel wall and there, in the presence of cytokines, differentiate into macrophages. These macrophages then take up oxidized LDL, which results in cholesteryl ester accumulation and "foam cell" formation, leading to early plaque formation. To determine the effect of SSW on these cell types in the heart blood vessels, tissue sections from the control and SSW exposed mice were immunolabeled with antibodies to F4/80, a marker for macrophages. Many more macrophages were present in the mice exposed to SSW smoke than in those breathing fresh air (Figure 6A, B). On the other hand, probing for neutrophils using antibodies to myeloperoxidase showed that these leukocytes are not present in the arterial vessel walls even in the mice exposed to SSW (Figure 6C, D), hence the effects of SSW are very specific for macrophages. These results support previous studies that showed that neutrophils were not implicated in atherosclerotic lesion development.

### Changes in adiponectin and TNFα levels in the plasma of SSW-exposed mice

**Figure 7:**
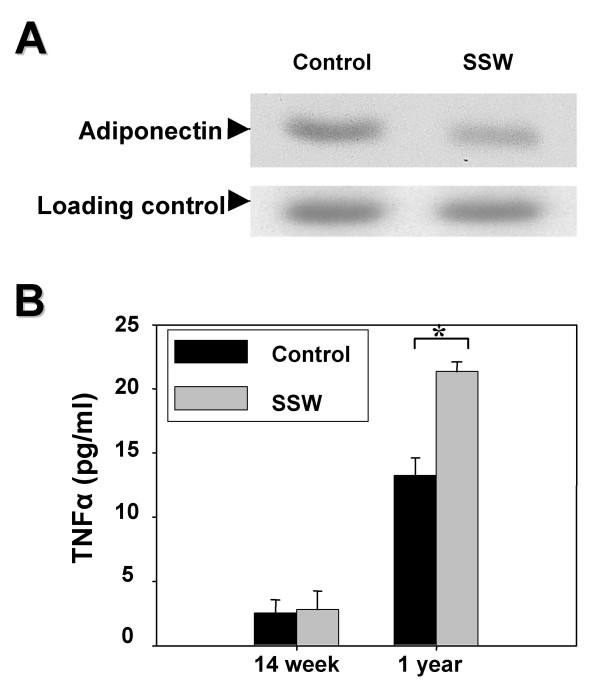
**Plasma levels of adiponectin and TNFα**. (**A**) Immunoblot analysis shows the levels of adiponectin in the plasma. Blood sample from both control and SSW smoking mice were applied to 7.5% polyacrylamide electrophoresis gel and transferred to the nitrocellulose membrane. Adiponectin was probed with a goat anti-mouse adiponectin antibody and with a secondary conjugated to HRP for subsequent use with the enhanced chemiluminescence system (ECL, Amersham Pharmacia). Less adiponectin was detected in the plasma of mice exposed to SSW. Samples representative of 2 animals per group. (**B**) Cytokine array results showing TNFα levels in the plasma. At early stages of smoking, there was no significant difference between the TNFα levels in the control and SSW smoked mice, whereas at late stages of SSW smoking, the TNFα level in plasma was significantly increased. This increment was correlated with the decrease of adiponectin and increment of MCP-1 in the plasma. Samples were done in triplicate.

Adiponectin is a protein primarily secreted by adipocytes that has potential anti-atherogenic properties [39-41]. The possibility that SSW exposure results in a decrease in the levels of circulating adiponectin was examined. Using immunoblot analysis and reducing conditions, it was found that the mice exposed to SSW had decreased plasma levels of adiponectin monomer (Figure 7A).

Production of MCP-1 and adiponectin are stimulated by TNFα [42-44]. At 14 weeks of smoking, there were no significant changes in the TNFα levels caused by exposure to SSW smoke. However, after 1 year of smoking, plasma TNFα levels were significantly increased compared to the control mice (Figure 7B).

### Changes in cytokine profiles in the plasma of SSW-exposed mice

**Figure 8:**
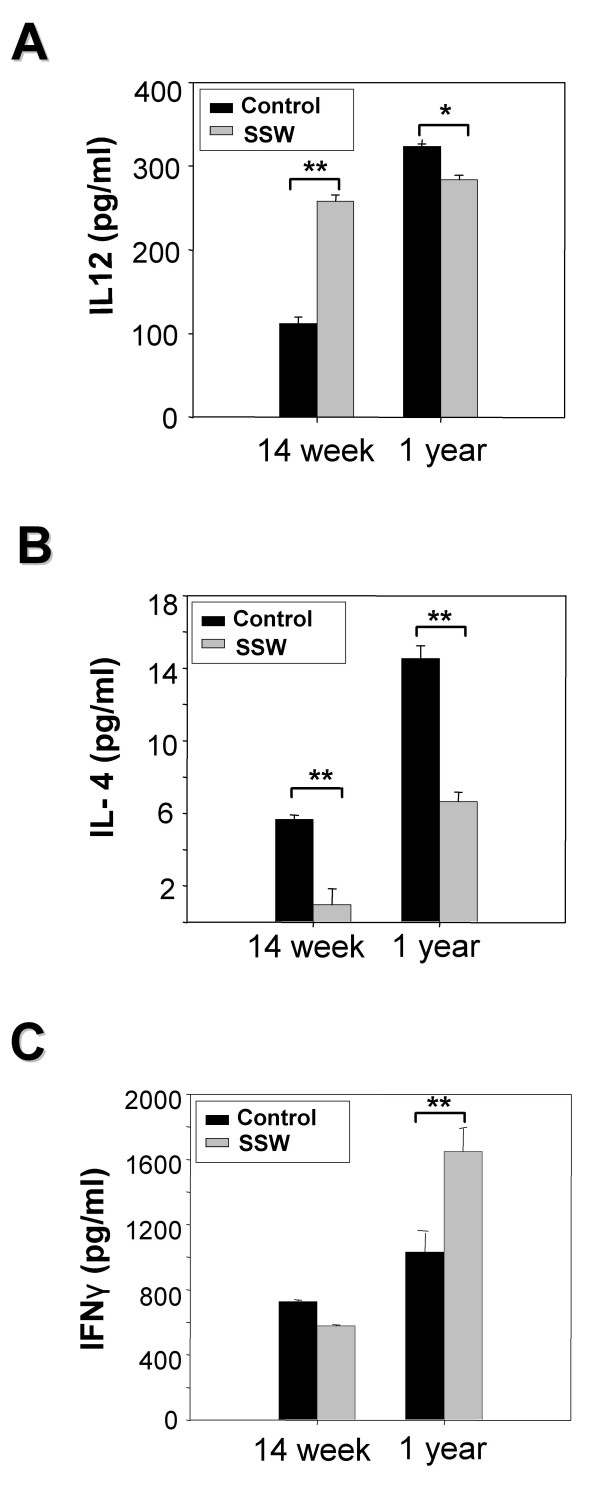
**Plasma levels of specific cytokines related to the immune response in atherosclerosis**. Cytokine array results show the changes of the cytokines IL-12 (**A**), IL-4 (**B**), and IFNγ (**C**) in the plasma of hApoB100 transgenic mice that were exposed to SSW smoke. The cytokines in the plasma were captured by antibodies coated on the bottom of 96-well plates (Quansys Biosciences, Utah, USA). After washing, probing with streptavidin-HRP, and reacting with substrate, the images were captured with CCD camera (Roper Micromax 1300B). The strength of signal was translated into concentration of cytokine by plotting against a standard curve.

**Figure 9:**
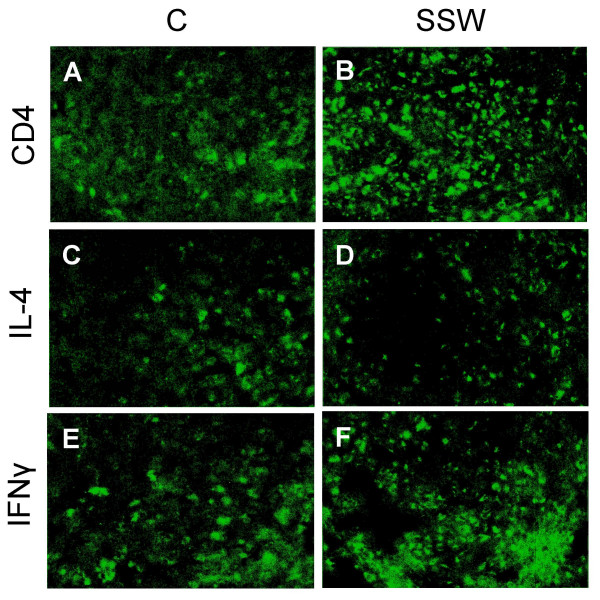
**SSW smoke exposure favors a Th1 immune response in human apoB100 Transgenic mice**. Tissue sections of the spleen were labeled with rat anti-mouse CD4+, IL-4, and IFNγ antibodies, and detected by FITC-labeled goat anti-rat IgG. (**A-F**) Immunolabeling showing the peripheral lymphocytes in spleen expressing CD4+ (**A, B**), IL-4 (**C, D**), and IFNγ **(E, F) **in hApoB100 transgenic mice that were exposed to normal air (**A, C, E**) or SSW smoke (**B, D, F**).

T-cell activation is an ongoing process in atherosclerotic lesions [45]. These immune cells can become activated and produce pro-inflammatory and cell-mediated responses (Th1 type), or produce cytokines that activate antibody-producing cells (Th2 type). Using a cytokine antibody array containing antibodies to detect Th1 and Th2 responses, we measured cytokine levels in the plasma of mice exposed to SSW. Cytokines such as IL-12 that promote Th1 responses and others such as IL-4 that promote Th2 responses were detected. It was found that at 14 weeks of SSW smoking, the plasma levels of the pro-inflammatory Th1-inducing IL-12 cytokine was significantly increased (Figure 8A) whereas IL-4, that promotes Th2 responses and antibody production, was significantly lower in the mice exposed to SSW smoke (Figure 8B). However, at 1 year of exposure to SSW, whereas the levels of IL-12 decreased slightly (Figure 8A), the levels of IL-4 remained sharply decreased (Figure 8B). Another critical cytokine required for Th1 immunity, IFNγ, also significantly increased at 1 year of exposure to SSW (Figure 8C). To further explore the possibility that Th1 cytokines remain elevated in the blood of smokers, we immunolabeled sections of spleen with antibodies to CD4, IL-4, and IFNγ γ and counted the number of cells that were positive for CD4, IL-4, and IFNγ. The results showed that the ratio of IFNγ to IL-4 was 2.82:1 in mice exposed to SSW, compared to 1.20:1 in control mice (Fig 9 and table 1). These results taken together suggest that smokers are in a pro-inflammatory state. That is, the normal switch from Th1 to Th2 response during immune system activation upon an insult, in this case circulating cigarette smoke components, does not occur.

**Table 1:** Number of splenic cells/HPF that express CD4+, IL-4 and IFNγ

	**Cell number**^a^			
		
**Treatment**	**CD4+**	**IL-4**	**IFNγ**	**IFNγ/IL-4**
**Control**	20.67 ± 4.49 (n = 3)*	27.00 ± 2.31 (n = 3)**	32.23 ± 2.33 (n = 3)	1.20
**SSW**	112.00 ± 4.62 (n = 3)^$, #, £^	33.33 ± 6.69 (n = 3)^§, †^	94.00 ± 7.81 (n = 3)^‡^	2.82

a, Splenic lymphocyte numbers were determined by immunolabeling of spleen sections with anti-CD4. The cell numbers in the clustered staining area was estimated by the size of the cells. P > 0.05 is considered not significant change, and p < 0.001 is considered very significant change. n represents the number of different high power fields within a tissue section.

*, Control/CD4+ vs. control/IL-4, and control/CD4+ vs. control/IFNγ: p > 0.05; **, control/IL-4 vs. control/IFNγ: p > 0.05, $, SSW CD4+ vs SSW IL-4: p < 0.001, and #, SSW CD4+ vs SSW/IFNγ: p > 0.05; § SSW IL-4 vs. SSW/IFNγ: p < 0.001 £, SSW CD4+ vs contol/CD4+: p < 0.001; †, SSW IL-4 vs. control/IL-4: p > 0.05; ‡, SSW/IFNγ vs control/IFNγ: p < 0.001 HPF = High Power Field

## Discussion

It is well known that cigarette smoking is detrimental to human health, leading to cardiovascular disease and cancer. Although cigarette smoke is widely accepted as an environmental factor that aggravates atherosclerosis [1,10-12], less well known are the biological processes by which cigarette smoke stimulates atherogenesis. By using a smoking system that greatly decreases the stress exerted on the animals, the effects of SSW smoke can be more accurately decoupled from effects of stress. Here we have shown that: (i) SSW smoke decreases circulating HDL and alters the physiology of other lipids, resulting in a significant increase in the area of the lesions found in the aorta and in more lipid deposition in the blood vessels of the heart; (ii) mice exposed to SSW have increased levels of MCP-1; (iii) macrophages accumulate in the arterial walls of mice exposed to cigarette smoke; (iv) adiponectin levels are decreased in the plasma of SSW smoking mice; (v) cigarette smoke stimulates accumulation of lipids in liver cells; and (vi) SSW stimulates cytokines that enhance the Th1 response (e.g. IL-12 and IFNγ) and suppresses cytokines that stimulate the Th2 response (e.g.IL-4).

Most humans suffering from atherosclerosis only have a moderately elevated cholesterol level, and have normal LDL receptors; hence the apoB100 transgenic mice used in this study may be a more physiologically relevant model for the study of atherosclerosis in most humans than other genetically modified mice. ApoB100 mice develop atherogenesis only on a relatively high fat diet, have moderately elevated cholesterol levels and have elevated levels of the apoB100 protein in their blood [22]. In general, humans that develop atherosclerosis have all three conditions; in particular they have elevated apoB100 [46-48] and consume high fat foods. All these features make human apoB100 transgenic mice the murine model of choice to study the "common" atherosclerotic disease state in humans exposed to cigarette smoke. As shown here, cigarette smoke increases lipid accumulation in blood vessels and induces more macrophage accumulation in the subendothelial layers of the aorta. These results correlate well with previous reports in which hyperlipidemic children were found to have low serum HDL levels when exposed to second-hand smoke [49]. The accumulation of macrophages in blood vessel walls and the presence of lipids are key steps in atherosclerotic plaque initiation.

Because MCP-1 functions in attracting monocytes, which migrate through the endothelial layer, differentiate into macrophages in the arterial walls and become activated and ingest oxidized lipids, it is quite possible that this molecule is a key modulator in the process of initiation of atherosclerosis plaque formation. For example, MCP-1 production is stimulated by balloon injury in rabbits [19] and pigs [20] and this stimulation is correlated with an increase in the number of monocytes and macrophages in the arterial wall. Furthermore, studies in apoE-/- mice show that these mice over-express MCP-1 and have accelerated atherosclerosis [21]. In contrast, knockout of either MCP-1 [22] or the MCP-1 receptor, CCR2, resulted in a marked reduction in atherosclerotic plaque formation [23]. Our data show that in mice exposed to SSW, the endothelial layer of arterial blood vessels produces higher levels of MCP-1, showing that this chemokine is in the right place at the right time to contribute to the initiation of plaque formation. Studies are under way to determine whether the MCP-1 increase is required for SSW-induced atherogenesis.

Artherosclerotic plaque formation may also be related to adipose tissue secreted molecules such as adiponectin. This is a 247 amino acid bioactive protein secreted by adipocytes [39]. Although adiponectin is an important modulator of glucose and lipid metabolism, recent studies found that adiponectin is potentially anti-atherogenic and anti-inflammatory [40,41]. In apo-E -/- mice, atherosclerotic plaque size decreased when adiponectin was overexpressed [50,51]. In patients with cardiovascular disease, plasma concentrations of adiponectin decreased in smokers [52,53]. Our data suggests that adiponectin is another modulator of cigarette-smoke-induced plaque formation. The low concentration of adiponectin in the blood of SSW-exposed mice shows that cigarette smoke can affect the levels of this protein in circulation. We are in the process of obtaining transgenic mice expressing both apoB100 and adiponectin to determine whether excess of this latter protein can decrease cigarette-smoke-induced atherogenesis.

MCP-1 is expressed by many different cell types upon stimulation of the cells by stress-inducing agents whereas adiponectin is constitutively expressed by adipocytes and is found at high concentrations (2–10 μg/ml) in the blood. Is the inverse expression level of MCP-1 and adiponectin important in SSW-induced atherosclerotic plaque formation? This connection may be critical for plaque formation and could be dependent on the cell-mediated immune responses to cigarette smoke components. As an important inflammatory cytokine, TNFα can upregulate MCP-1 expression [53] and down-regulate adiponectin expression [44]. In SSW-exposed mice, the MCP-1 levels at 14 weeks of smoking correlate well with the levels of TNFα at this same time. Furthermore, after 1 year of SSW exposure, when the levels of TNFα are significantly increased, the MCP-1 levels are also significantly increased whereas adiponectin levels are significantly decreased. Therefore, on the one hand, cigarette smoke increased the levels of TNFα thereby causing MCP-1 expression by the endothelial cells of the aorta to attract monocytes, which led to accumulation of oxidized lipids in the subendothelial layer. However, on the other hand, increased levels of TNFα induced by cigarette smoke also decreased the circulating levels of adiponectin, which limited its function in protecting the endothelial layer. These two processes thus may both contribute to enhancing the progression of the initiation of atherosclerotic plaque formation.

T-cells are known to be present in atherosclerotic lesions, suggesting that they are important in cell-mediated immune responses during atherogenesis. Most of these T cells are CD 4^+ ^T cells (Th type). The pro-inflammatory cytokine IL-12, the center for Th1 immunity [54], stimulates Th1 cell differentiation and expression of other cytokines, such as TNFα and IFNγ [55]. IL-12 levels were significantly increased at the early stage of SSW exposure, and this correlated well with the significant increase in the levels of TNFα over time. There was no significant change in the level of IL-12 for the mice exposed to SSW, meaning that these mice are constantly stimulated to produce this cytokine by SSW components. These data show that SSW can stimulate IL-12 and IFNγ expression and thus enhance the Th1 response in atherogenic lesion formation. On the other hand, SSW also suppress the expression IL-4, a Th2 stimulating cytokine indicating that the switch from a pro-inflammatory Th1 response to an antibody-dependent Th2 response is impaired.

In addition to all of these effects, cigarette smoke also causes hepatic steatosis (fatty liver), which in clinical studies correlates with atherogenesis. Although there are no direct studies regarding the relationship between cigarette smoking and hepatic steatosis, it has been reported that cigarette smoking can increase severity of hepatic lesions in patients with chronic hepatitis C [56,57]. Starting smoking was also associated with deterioration of serum alanine aminotransferase (ALT) in NAFLD patients [58]. One potential complication in interpreting our results on cigarette smoke-induced steatosis is that apoB100 transgenic mice require high fat diet to induce lesion development in the aorta in a reasonable time frame. The high fat diet contains cholate, which is known to cause liver dysfunction and inflammation. However, we believe that our results show true effects of cigarette smoke because, although the control groups were not exposed to SSW, they were fed the same diet as the mice that were exposed to SSW. Therefore, the difference in lipid metabolism can be attributed to the effect of cigarette smoke. However, the possibility remains that some of the effects are due to an interaction between smoke exposure and dietary cholate.

## Conclusion

Our results suggest that the levels of both MCP-1 and adiponectin are regulated by the cytokine TNFα, which is modulated by IL-12. Because modulation of both MCP-1 and adiponectin plays important functions in atherosclerosis, the imbalance of these two molecules may be key to atherogenesis. Our results also suggest that SSW inhibits the switch of Th1 to Th2 response, leading to an imbalance of the Th response, thereby linking the importance of the immune response to the process of second-hand smoke-induced atherogenesis and suggesting that smokers may live in a permanent state of inflammation.

## Abbreviations

ApoB Apolipoprotein B

MSW MainStream Whole

SSW SideStream Whole

MCP-1 monocyte chemoattractant protein–1

LDL low density lipopotein

ApoE apoliprotiein E

LDLR low density lipopotein receptor

TPM total particulate matter

TG triglyceride

TC total cholesterol

IL interleukin

TNFα tumor necrosis factor alpha

VCAM vascular adhesion molecules

ICAM intercellular adhesion molecules

## Competing interests

Part of the funding to carry out the studies presented in this manuscript came from the Philip Morris External Research Program.

## Authors' contributions

HY carried out all studies except for carboxyhemoglobin measurement. LW conceived the smoking system and animal model selection and participated in system calibration and cholesterol measurement. MB and MZ carried out carboxyhemoglobin measurement. CM helped to smoke the mice and to process the tissues. MS and REP supplied the human apoB 100 transgenic mice of 50% C57 black/50% SJL background. MMG conceived and designed the studies with HY and LW, and contributed to manuscript preparation and writing. YM did the cytokine array experiment. All authors read and approved the final manuscript.

## Pre-publication history

The pre-publication history for this paper can be accessed here:


